# Delayed low cerebellar perfusion status is associated with poor outcomes in top-of-basilar occlusion treated with thrombectomy

**DOI:** 10.3389/fneur.2023.1161198

**Published:** 2023-04-25

**Authors:** Jae-Chan Ryu, Boseong Kwon, Yunsun Song, Deok Hee Lee, Jun Young Chang, Dong-Wha Kang, Sun U. Kwon, Jong S. Kim, Bum Joon Kim

**Affiliations:** ^1^Department of Radiology, Asan Medical Center, University of Ulsan College of Medicine, Seoul, Republic of Korea; ^2^Department of Neurology, Asan Medical Center, University of Ulsan College of Medicine, Seoul, Republic of Korea; ^3^Department of Neurology, Gangneung Asan Hospital, University of Ulsan College of Medicine, Gangneung, Republic of Korea

**Keywords:** mechanical thrombectomy, top-of-basilar artery occlusion, perfusion, lower cerebellum, endovascular treatment (EVT)

## Abstract

**Background and purpose:**

Top-of-basilar artery occlusion (TOB) is one of the most devastating strokes despite successful mechanical thrombectomy (MT). We aimed to investigate the impact of initial low cerebellum perfusion delay on the outcomes of TOB treated with MT.

**Methods:**

We included patients who underwent MT for TOB. Clinical and peri-procedural variables were obtained. Perfusion delay in the low cerebellum was defined as (1) time-to-maximum (Tmax) >10 s lesions or (2) relative time-to-peak (rTTP) map >9.5 s with a diameter of ≥6 mm in the low cerebellum. The good functional outcome was defined as the achievement of a modified Rankin Scale score of 0–3 at 3 months after stroke.

**Results:**

Among the 42 included patients, 24 (57.1%) patients showed perfusion delay in the low cerebellum. The admission National Institutes of Health Stroke Scale (NIHSS) score was significantly higher in those with perfusion delay [17 (12–24) vs. 8 (6–15), *P* = 0.002]. Accordingly, the proportion of good functional outcomes was lower in those with perfusion delay than in those without [5 (20.8%) vs. 13 (72.2%), *P* = 0.003]. From the multivariable analysis, the admission NIHSS score [odds ratio (OR) = 0.86, 95% confidence intervals (CIs) = 0.75–0.98, *P* = 0.021] and low cerebellum perfusion delay (OR = 0.18, 95% Cis = 0.04–0.86, *P* = 0.031) were independently associated with the 3-month functional outcomes.

**Conclusion:**

We found that initial perfusion delay proximal to TOB in the low cerebellum might be a predictor for poor functional outcomes in TOB treated with MT.

## Introduction

Top-of-basilar artery occlusion (TOB) is one of the most catastrophic strokes (1). Mechanical thrombectomy (MT) has been widely used in real-world practice, despite limited evidence of the efficacy of reperfusion treatment for posterior circulation large-vessel occlusion *(LVO)* (2–4). For the proper selection of candidates, a recent study showed that multimodal magnetic-resonance (MR) imaging, including perfusion imaging, was useful for predicting functional outcomes after basilar artery (BA) thrombectomy (5).

Theoretically, TOB can cause ischemic damage with a penumbra distal to the occlusion site, mostly in the posterior cerebral artery (PCA) and superior cerebellar artery (SCA) territory. Previous studies have shown that cerebral perfusion is affected by the inflow from the artery and the outflow through the vein (6, 7). Moreover, recent studies have shown that the dysfunction of outflow in LVO is associated with elevated venous pressure and brain edema and is consequently associated with the functional outcome of stroke (8, 9).

In real-world practice, clinicians often encounter patients with TOB who show delayed perfusion proximal to the TOB in the territories of the anterior or posterior inferior cerebellar artery (AICA or PICA) before MT. We hypothesized that unlike in the anterior circulation LVO, such as middle cerebral artery occlusion, which has an alternative flow path including the anterior cerebral artery, the blood flow stasis, and congestion proximal to the occlusion site, may more easily occur in TOB as it is an occlusion of the single path end-artery (10, 11). Therefore, we investigated that initial perfusion delay proximal to TOB at the low cerebellum would be associated with the outcome in patients treated with MT.

## Methods

### Study population and data collection

Patients who underwent MT for LVO within 24 h of symptom onset between January 2012 and July 2022 were retrospectively included. Among them, patients with ischemic stroke due to anterior circulation LVO and those who did not have TOB (lower-, or mid-BA occlusion) were excluded. Baseline characteristics, clinical factors, and procedure-related factors, including onset-to-puncture time, diffusion-restricted lesion volume before MT, modified thrombolysis in cerebral infarction (mTICI) grade, first-pass effect, and the MT method (contact aspiration, stent-retriever, or angioplasty), were obtained. mTICI grade 2b/3 was defined as successful recanalization. The patients routinely underwent *gradient-echo imaging* 1 day after MT for the detection of hemorrhagic transformation. The main outcome was the achievement of a modified Rankin Scale (mRS) score of 0–3 (good functional outcome) at 3 months (12, 13). Informed consent was waived due to its retrospective nature. The local ethics committee of our tertiary hospital approved this study (No. 2022-1425).

### Perfusion delay in the low cerebellum

**Figure 1:**
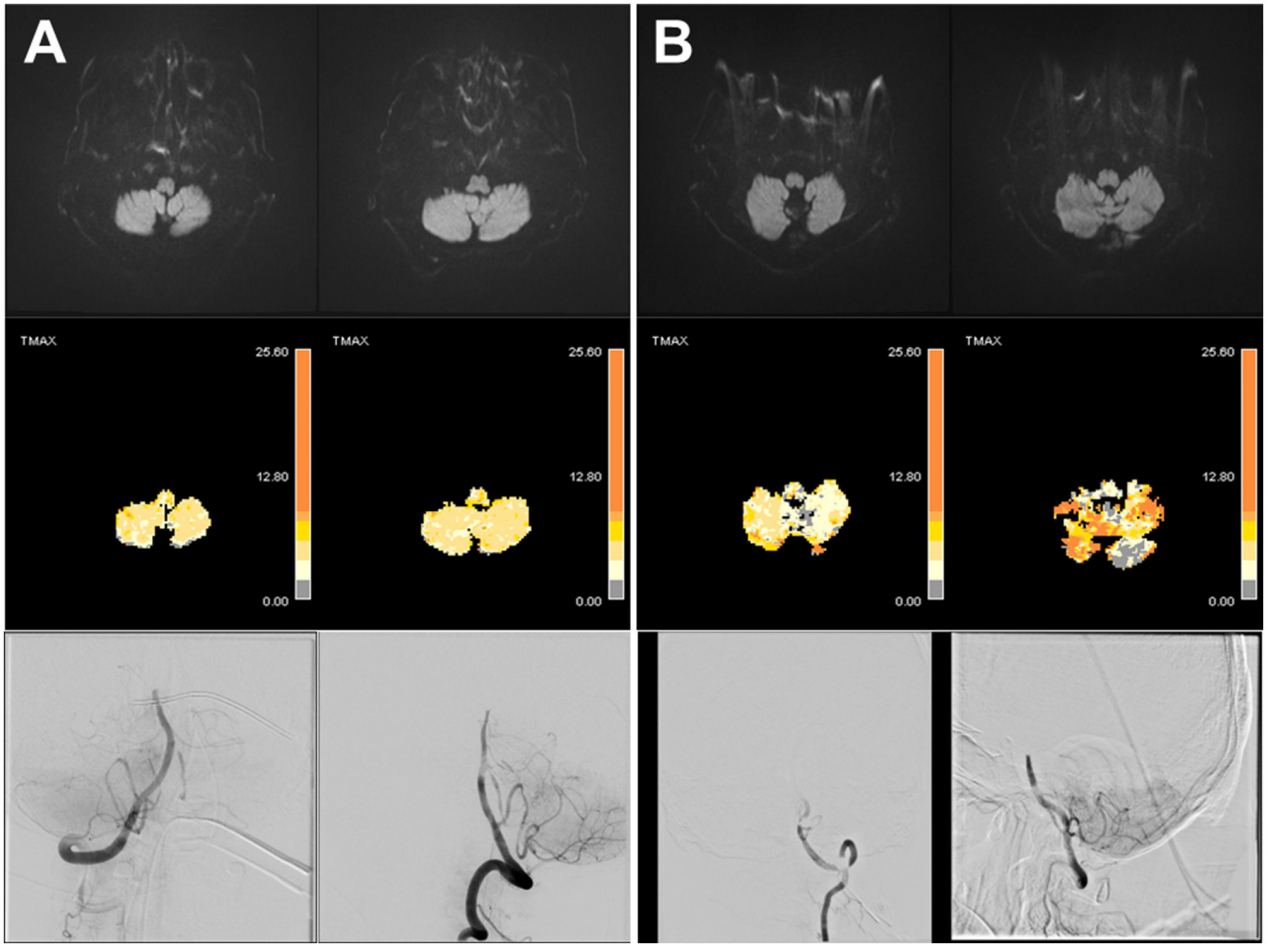
Representative cases of top-of-basilar occlusion with or without perfusion delay in the low cerebellum. There were two cases of top-of-basilar artery occlusion without diffusion-restriction lesions. There was no perfusion delay in the low cerebellum **(A)**; however, there was a prominent perfusion delay in the low cerebellum **(B)**.

The patients underwent diffusion- and perfusion-weighted imaging before MT. Because our institution has been using OLEA software (Olea Medical Solutions, La Ciotat, France) since 2017, we defined perfusion delay in the low cerebellum as the following two definitions. From 2017 to 2022, time-to-maximum (Tmax) >10 s lesions in OLEA software were defined as delayed perfusion in the low cerebellum (Figure 1) (5). From 2012 to 2016, a relative time-to-peak (rTTP) map > 9.5 s lesions in the rTTP map were defined as delayed perfusion in the low cerebellum (14). All rTTP map imaging measurements were performed on a Siemens 1.5 T scanner (Siemens Avanto 1.5T, Erlangen, Germany). Only the lesions with a diameter of 6 mm or more in AICA or PICA territory were recognized as perfusion delay in the low cerebellum (5).

### Statistical analysis

The comparison of variables according to the presence of perfusion delay in the low cerebellum was assessed using the Student's *t*-test, Mann–Whitney *U*-test, and chi-square test, as appropriate. We calculated odds ratios (ORs) and 95% confidence intervals (CIs) using univariable logistic regression analysis for a good functional outcome. Factors with a significant association (*P* < 0.05) in the univariable analysis were included in the multivariable analysis. In this study, a *P*-value of < 0.05 was considered to be statistically significant. All of the analyses were performed using R Software (version 4.2.0; R Foundation for Statistical Computing, Vienna, Austria).

## Results

During the study period, 801 patients underwent MT for LVO. Among them, 695 patients who underwent MT for anterior circulation LVO and 64 patients who underwent MT for vertebral artery occlusion, lower-, or mid-BA occlusion were excluded. Finally, a total of 42 patients with TOB were included (Supplementary Figure S1). The mean age of the patients was 70.3 ± 11.2 years old, and 23 (54.8%) were men. We analyzed 33 patients (78.6%) via the OLEA and nine patients (21.4%) via the rTTP map. Among the patients, 24 (57.1%) showed initial perfusion delay in the low cerebellum, proximal to the TOB.

The baseline National Institutes of Health Stroke Scale (NIHSS) score was significantly higher in patients with perfusion delay than in those without perfusion delay in the low cerebellum [17 (12–24) vs. 8 (6–15), *P* = 0.002; Table 1]. The mRS score at 3 months was significantly higher [5.0 (4.0–5.0) vs. 2.0 (1.0–4.0), *P* = 0.001], and the proportion of good functional outcomes was significantly lower in those with perfusion delay [5 (20.8%) vs. 13 (72.2%), *P* = 0.003; Supplementary Figure S2]. There was a trend showing a higher proportion of hemorrhagic transformation after MT in those with perfusion delay [12 (50.0%) vs. 3 (16.7%), *P* = 0.057].

**Table 1:** Characteristics according to the perfusion delay in the low cerebellum.

	**Perfusion delay (–) (*N* = 18)**	**Perfusion delay (+) (*N* = 24)**	***P***
Age	67.8 ± 13.1	72.1 ± 9.3	0.222
Male sex	7 (38.9)	16 (66.7)	0.140
Onset-to-puncture time, min	489 [207–705]	643 [315–1,004]	0.347
Hypertension	10 (55.6)	13 (54.2)	>0.999
Diabetes	6 (33.3)	8 (33.3)	>0.999
Hyperlipidemia	7 (38.9)	4 (16.7)	0.205
Atrial fibrillation	11 (61.1)	12 (50.0)	0.687
Smoking	4 (22.2)	12 (50.0)	0.130
Stroke history	5 (27.8)	6 (25.0)	>0.999
Intravenous thrombolysis	6 (33.8)	5 (20.8)	0.577
Baseline mRS	0.0 [0.0–1.0]	0.0 [0.0–1.0]	0.685
Admission NIHSS	8 [6–15]	17 [12–24]	0.002
3-month mRS	2.0 [1.0–4.0]	5.0 [4.0–5.0]	0.001
mRS 0–3	13 (72.2)	5 (20.8)	0.003
Mortality	0 (0.0)	4 (16.7)	0.197
Stroke etiology			0.517
Large-artery disease	2 (11.1)	6 (25.0)	
Cardioembolism	12 (66.7)	13 (54.2)	
Others	4 (22.2)	5 (20.8)	
Ischemic core, cc	4.1 [0.9–8.3]	5.8 [3.1–19.4]	0.166
Successful recanalization	17 (94.4)	20 (83.3)	0.536
First-pass effect	8 (50.0)	14 (53.8)	>0.999
Hemorrhagic transformation	3 (16.7)	12 (50.0)	0.057
MT method			
Contact aspiration	11 (61.1)	19 (79.2)	0.349
Stent-retriever	7 (38.9)	9 (37.5)	>0.999
Angioplasty	2 (11.1)	4 (16.7)	0.949

Values are expressed as number (%), mean ± standard deviation, and median [interquartile range].

NIHSS, National Institutes of Health Stroke Scale; mRS, modified Rankin Scale; MT, mechanical thrombectomy.

In the univariable logistic regression analysis, low admission NIHSS score (OR = 0.84, 95% Cis = 0.75–0.94, *P* = 0.003) and absence of perfusion delay in the low cerebellum (OR = 0.10, 95% Cis = 0.02–0.42, *P* = 0.002) had significant associations with a good functional outcome (Table 2). From the multivariable analysis, low admission NIHSS score (OR = 0.86, 95% Cis = 0.75–0.98, *P* = 0.021) and absence of perfusion delay in the low cerebellum (OR = 0.18, 95% Cis = 0.04–0.86, *P* = 0.031) were independently associated with a good functional outcome.

**Table 2:** Factors associated with mRS scores of 0–3.

**Variable**	**cOR (95% CI)**	***P***	**aOR (95% CI)**	***P***
Age	0.95 (0.90–1.01)	0.128		
Male sex	0.32 (0.09–1.14)	0.078		
Onset-to-puncture time	1.000 (0.999–1.001)	0.439		
Hypertension	0.71 (0.21–2.44)	0.592		
Diabetes	2.40 (0.65–8.90)	0.191		
Hyperlipidemia	3.18 (0.76–13.3)	0.113		
Coronary artery disease	1.36 (0.29–6.38)	0.914		
Atrial fibrillation	1.57 (0.45–5.43)	0.475		
Smoking	0.29 (0.07–1.12)	0.073		
Stroke history	1.15 (0.29–4.61)	0.840		
Intravenous thrombolysis	0.69 (0.17–2.86)	0.613		
Admission NIHSS	0.84 (0.75–0.94)	0.003	0.87 (0.78–0.98)	0.021
Stroke etiology				
Non-cardioembolism	Reference			
Cardioembolism	1.69 (0.48–6.01)	0.416		
Ischemic core	0.90 (0.80–1.01)	0.071		
Successful recanalization	N/A	N/A		
First-pass effect	1.86 (0.54–6.43)	0.329		
Hemorrhagic transformation	0.34 (0.09–1.33)	0.121		
Contact aspiration	0.67 (0.17–2.56)	0.555		
Stent-retriever	1.60 (0.45–5.63)	0.464		
Angioplasty	3.14 (0.51–19.5)	0.219		
Perfusion delay in low cerebellum	0.10 (0.02–0.42)	0.002	0.18 (0.04–0.86)	0.031

mRS, modified Rankin Scale; cOR, crude odds ratio; aOR, adjusted odds ratio; NIHSS, National Institutes of Health Stroke Scale; N/A, not available.

## Discussion

In the current study, we focused on the initial perfusion delay in the low cerebellum in stroke patients treated with MT for TOB, which has rarely been investigated. Patients with perfusion delay in the low cerebellum had severe initial stroke severity and a higher proportion of poor functional outcomes at 3 months. Moreover, perfusion delay in the low cerebellum in TOB before MT was independently associated with the poor functional outcome of stroke.

TOB may have increased distal resistance, reducing the blood flow in the proximal area of occlusion in the low cerebellum and leading to higher initial stroke severity with poor functional outcomes. However, not only the inflow of arterial blood to the brain but also the venous outflow can affect the outcome of stroke. Previous studies found that venous outflow associated with perfusion delay could be a predictor of the outcome in patients with MT (8, 15–17).

**Figure 2:**
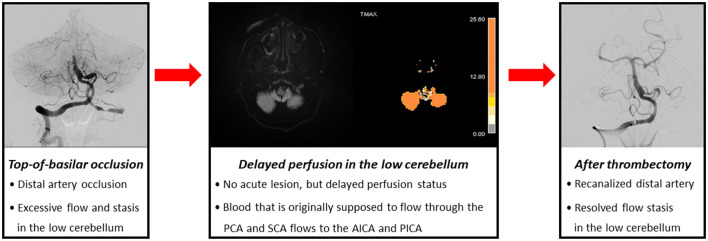
Hypothesis regarding excessive blood flow and stasis proximal to the occlusion site in top-of-basilar occlusion.

In patients with TOB, delayed perfusions in PCA and SCA territories could be well understood. However, it is difficult to explain the perfusion delay in AICA and PICA territories in TOB. Theoretically, blood that is originally supposed to flow through the PCA and SCA flows to the AICA or PICA, causing congestion due to sudden excessive blood flow in the low cerebellum, and consequently, dysfunction of venous drainage in the low cerebellum (Figure 2). Previous studies have shown that outflow stasis in LVO can cause elevated venous pressure and aggravation of brain edema and can affect the functional outcome of stroke. Therefore, perfusion delay in the low cerebellum might reflect congestion in the outflow stasis, as in venous infarction (11). Similarly, the proportion of patients with hemorrhagic transformation was slightly more frequent in those with perfusion delay. More than half of the patients with hemorrhagic transformation in the delayed cerebellar perfusion group (7 out of 12 patients) showed hemorrhagic transformation in the AICA or PICA territory, where there was no initial ischemic lesion but delayed perfusion. Therefore, delayed perfusion with blood flow congestion in AICA and PICA territories may have caused blood–brain barrier disruption, and this might have induced hemorrhagic transformation with the recanalization of TOB.

This study has some limitations. First, our study was a retrospective study with selection bias, and only a small number of patients were included due to the scant number of patients with TOB. Therefore, the generalizability is limited, and our findings require cautious interpretation. Second, perfusion delay was defined using two different methods, although a previous study has shown that the two definitions correspond well to each other (14). Third, we could not exclude the possibility that AICA or PICA was transiently occluded by an embolic source and spontaneously recanalized.

Despite these limitations, we suggested that initial perfusion delay in the low cerebellum could be a predictor for poor functional outcomes treated with MT for TOB. The mechanism of this association might be outflow congestion due to sudden excessive blood flow in TOB.

## Data availability statement

The raw data supporting the conclusions of this article will be made available by the authors, without undue reservation.

## Ethics statement

The studies involving human participants were reviewed and approved by the Local Ethics Committee of Asan Medical Center (No. 2022-1425). The Local Ethics Committee of Asan Medical Center waived the written informed consent because this study was retrospective design. Written informed consent for participation was not required for this study in accordance with the national legislation and the institutional requirements.

## Author contributions

J-CR contributed to the study concept and design, data collection and interpretation, draft preparation, and revision of the manuscript. BKw, YS, DL, JC, D-WK, SK, and JK contributed to the data interpretation and revision of the manuscript. BKi contributed to the study concept and design, data interpretation, and draft preparation and revision of the manuscript. All authors contributed to the article and approved the submitted version.
