# Supplementary material for: Delayed low cerebellar perfusion status is associated with poor outcomes in top-of-basilar occlusion treated with thrombectomy
Source: Front Neurol. 2023 Apr 25;14:1161198. doi: 10.3389/fneur.2023.1161198 (PMC10166797; doi:10.3389/fneur.2023.1161198)

**Supplemental Materials**

**Figure S1.** Flow diagram of the patients included in this study


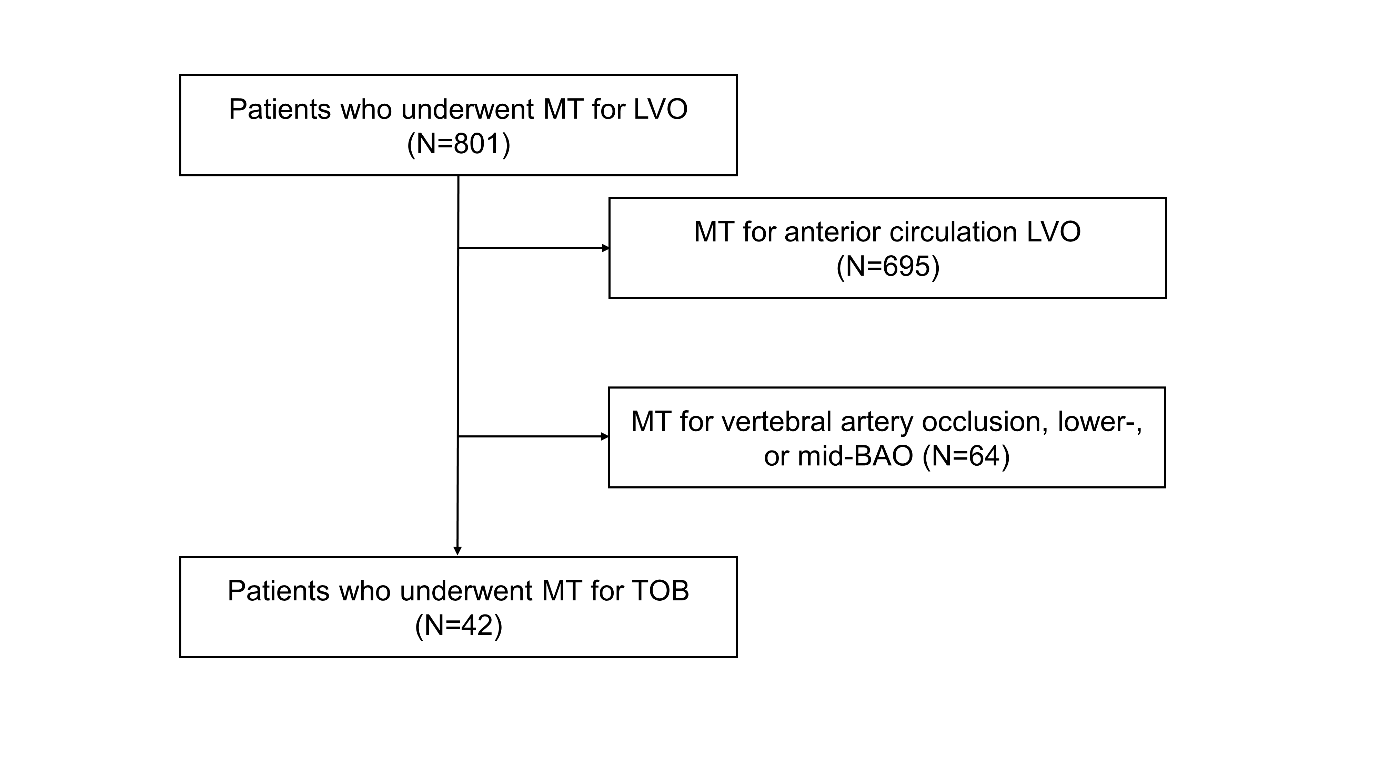


MT, mechanical thrombectomy; LVO, large-vessel occlusion; TOB, top-of-basilar artery occlusion.

**Figure S2.** Distribution of modified Rankin Scale scores at 3 months after mechanical thrombectomy for top-of-basilar occlusion according to the presence of perfusion delay in the low cerebellum.


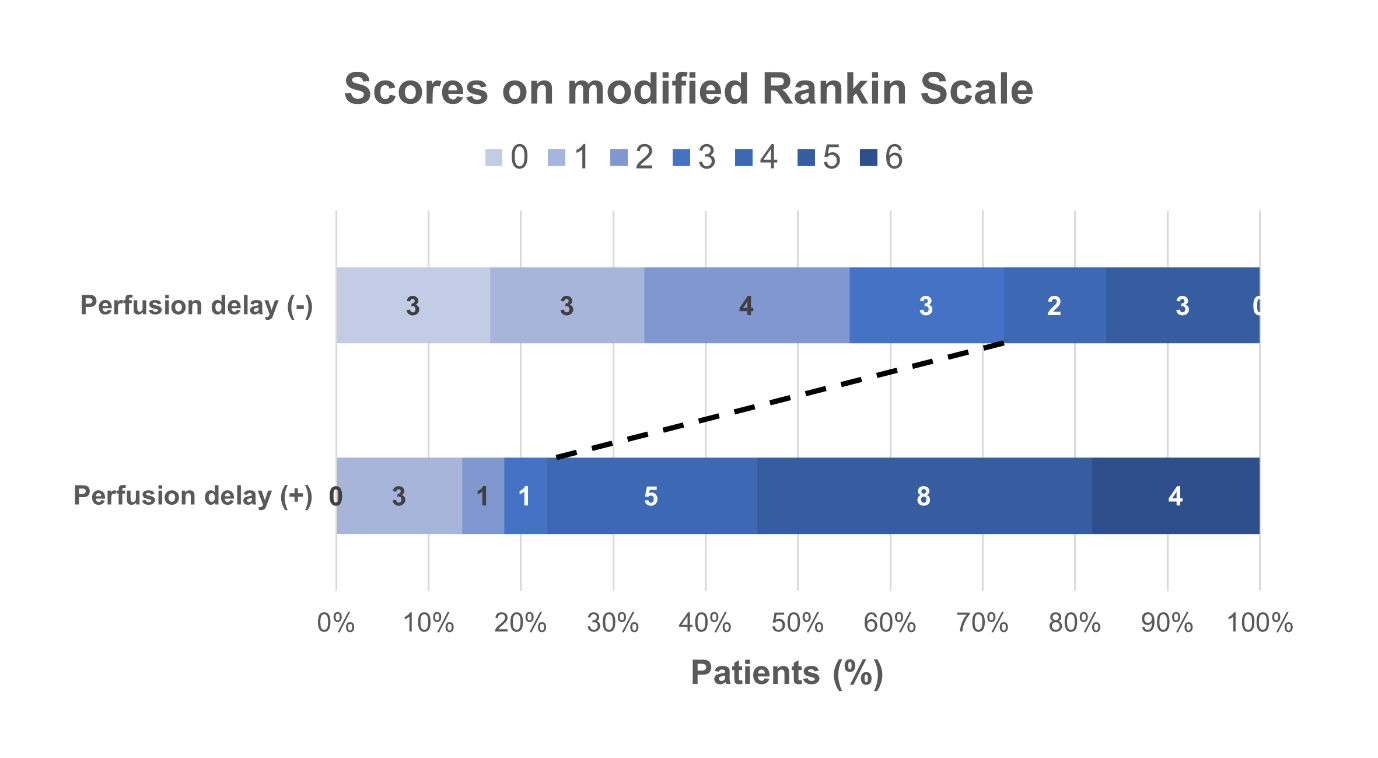

Supplement: Supplementary file 1 [file Data_Sheet_1.docx]
